# Misclassification of TP53 germline variants: implications for survival analysis in AML transplant studies

**DOI:** 10.1038/s41375-025-02772-7

**Published:** 2025-09-28

**Authors:** Léa Rodriguez, François Delhommeau, Thierry Soussi

**Affiliations:** 1 Sorbonne Université, INSERM, Centre de Recherche Saint-Antoine, CRSA, UMRS_938 Hematopoietic and Leukemic Development, AP-HP, SIRIC CURAMUS, Paris, France; 2 Carnot OPALE Institute, Paris, France; 3 Department of Immunology, Genetics and Pathology, Laboratory, Uppsala University, Uppsala, Sweden

**Keywords:** Cancer, Cancer genetics

## To the Editor:

We read with interest the recent work by Zhao et al. on the impact of *TP53* variants and germline mutations on allogenic stem cell transplantation outcomes in acute myeloid leukemia (AML) [1].

Allogenic stem cell transplantation remains a cornerstone of curative therapy for patients with AML, offering the potential for long-term remission and survival. *TP53* mutations in AML are associated with poor prognosis, reduced response rates to conventional therapies and high relapse risks even after allogeneic stem cell transplantation. Evaluating *TP53* status as described in the study by Zhao et al. is therefore essential, as it remains a key independent predictor of adverse outcomes and influences both patient selection and post-transplant management strategies. In their study, the authors compared the impact of somatic versus germline *TP53* mutations on patient outcomes. They concluded that patients with germline mutations had significantly better outcomes compared to patients with somatic mutations. Although the landscape of p53 somatic *TP53* variants shows that these are mostly well-known pathogenic variants, this is not the case for the germline variants described by the authors. Among the 20 cases described in their Fig. S1, five (25%) variants (particularly rs201753350, p.V31I) are indeed genuine non-pathogenic SNPs specific to the Asian population.

**Fig. 1:**
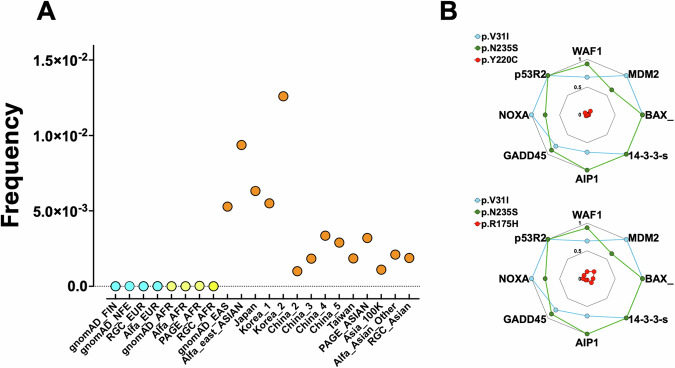
Frequency and functional activity of rs201753350 (p.V31I). **A** The sources of the different populations are described in [2]. rs201753350 is highly specific to the East Asian population and is not found in other populations. **B** Functional analysis of rs201753350 (p.V31I) compared to p.N235S, a benign p53 SNP and p.R175H and p.Y220C, two nonfunctional hotspot variants found in AML and described as somatic mutations in the study of Zhao et al. The remaining activity (functional data on eight p53 response elements) ranges from 0 (no activity) to 1 (full activity) and was defined from the normalized data of Kato et al. [4].

Historically, rs1042522 (p.P72R) was the first missense SNP described for *TP53*, but more recent studies, using various population databases, have identified more than 20 novel nonpathogenic missense SNPs, including ethnic-specific variants [2]. Among them are several variants specific to the Asian population, including rs201753350 (p.V31I) (Fig. 1A). Functional analysis shows that this variant is not impaired (Fig. 1B) [3–5]. Multiple Asian studies have shown that variant rs201753350 is not associated with an increased risk of cancer [6, 7]. Unfortunately, the ClinVar classification of this variant is still conflicting. Therefore, mixing both genuine benign SNPs and true pathogenic variants may suggest a serious bias in the various survival analyses performed by the authors.

Most SNP databases, including resources like dbSNP or GnomAD, continue to demonstrate significant ethnocentrism, as their data predominantly represent individuals of European ancestry. This leads to population bias in genetic analyses [8]. As shown in the study by Zhao et al., this bias can hinder the identification of relevant genetic variants in underrepresented populations, affecting both biomedical research and the accuracy of disease association studies. Dedicated efforts have identified and cataloged ethnically variable SNPs, but gaps in truly global representation persist. Ongoing initiatives now aim to address this imbalance by diversifying ancestry in sample collection, but most reference datasets still reflect historical and structural biases toward Western populations [9].
